# VEGFR2 Trafficking, Signaling and Proteolysis is Regulated by the Ubiquitin Isopeptidase USP8

**DOI:** 10.1111/tra.12341

**Published:** 2015-12-02

**Authors:** Gina A. Smith, Gareth W. Fearnley, Izma Abdul‐Zani, Stephen B. Wheatcroft, Darren C. Tomlinson, Michael A. Harrison, Sreenivasan Ponnambalam

**Affiliations:** ^1^ Endothelial Cell Biology Unit, School of Molecular & Cellular BiologyUniversity of LeedsLS2 9JTLeedsUK; ^2^ Leeds Institute of Cardiovascular & Metabolic MedicineUniversity of LeedsLS2 9JTLeedsUK; ^3^ Biomedical Health Research Centre & Astbury Centre for Structural Molecular BiologyUniversity of LeedsLS2 9JTLeedsUK; ^4^ School of Biomedical SciencesUniversity of LeedsLS2 9JTLeedsUK

**Keywords:** de‐ubiquitination, proteolysis, signal transduction, trafficking, USP8, VEGF‐A, VEGFR2

## Abstract

Vascular endothelial growth factor A (VEGF‐A) regulates many aspects of vascular function. VEGF‐A binding to vascular endothelial growth factor receptor 2 (VEGFR2) stimulates endothelial signal transduction and regulates multiple cellular responses. Activated VEGFR2 undergoes ubiquitination but the enzymes that regulate this post‐translational modification are unclear. In this study, the de‐ubiquitinating enzyme, USP8, is shown to regulate VEGFR2 trafficking, de‐ubiquitination, proteolysis and signal transduction. USP8‐depleted endothelial cells displayed altered VEGFR2 ubiquitination and production of a unique VEGFR2 extracellular domain proteolytic fragment caused by VEGFR2 accumulation in the endosome–lysosome system. In addition, perturbed VEGFR2 trafficking impaired VEGF‐A‐stimulated signal transduction in USP8‐depleted cells. Thus, regulation of VEGFR2 ubiquitination and de‐ubiquitination has important consequences for the endothelial cell response and vascular physiology.

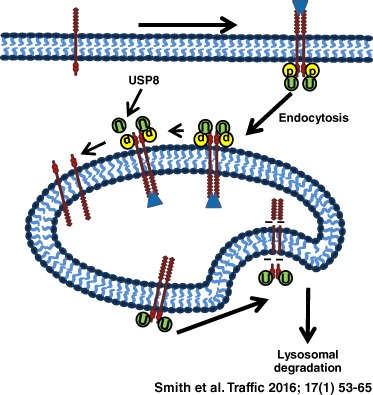

Vascular endothelial growth factor‐A (VEGF‐A) is a potent pro‐angiogenic growth factor involved in regulating angiogenesis 1. VEGF exerts its effects by binding and activating a family of three vascular endothelial growth factor receptor tyrosine kinases (VEGFRs), namely VEGFR1, VEGFR2 and VEGFR3. VEGFR2 (KDR) is the principal receptor through which VEGF‐A transmits its pro‐angiogenic signals in vascular endothelial cells 2, 3. VEGF‐A binding to VEGFR2 promotes dimerization and trans‐autophosphorylation of several key tyrosine residues present within its cytoplasmic kinase domain 1. Upon activation, VEGFR2 enters the endosome–lysosome system through incorporation into clathrin‐coated vesicles and trafficking to early endosomal vesicular compartments 4.

Ubiquitination of VEGFR2 acts as an endosomal sorting signal by binding to the ubiquitin‐interacting motif of ESCRT‐0 components, Hrs and STAM 4, 5, 6. Internalized VEGFR2 can recycle back to the plasma membrane or be committed for lysosomal degradation 6, 7. Ubiquitination is a dynamic protein modification that coordinates receptor trafficking, recycling and degradation 8. Reversibilty of ubiquitination is credited to the action of de‐ubiquitinating enzymes (DUBs) 8. These enzymes thus play a distinct but crucial role in receptor tyrosine kinase trafficking and turnover 8.

DUBs are a superfamily of 91 enzymes that can be subdivided into five distinct subfamilies with differing specificities for the isopeptide bond that links ubiquitin chains 9. De‐ubiquitination of plasma membrane receptors facilitates recycling and is essential for maintaining the free ubiquitin pool upon which receptor trafficking is dependent. Similar to the co‐ordinated but opposing effects of kinase and phosphatase activity, ubiquitination is kept in balance by the activity of DUBs 10.

Although it is known that VEGFR2 is recycled from endosomes back to the plasma membrane, it is unknown which DUBs prevent its lysosomal degradation. Ubiquitin‐specific protease Y (UBPY or USP8) is a DUB be involved in the trafficking of epidermal growth receptor tyrosine kinase (EGFR) 11, 12, 13. USP8 is a cysteine protease and member of the ubiquitin‐specific protease (UBP) family of DUB enzymes capable of catalyzing complete breakdown of both K48‐ and K63‐linked polyubiquitin into its component monomers 11, 14, 15. USP8 has diverse roles in membrane trafficking ranging from endosomal regulation to retrograde transport 11, 13. The early endosome ESCRT‐0 subunit, STAM, is a USP8‐binding partner 16, 17. This interaction occurs via the SH3 domain of STAM and the proline‐rich STAM‐binding motif in USP8 17. USP8 depletion inhibits EGFR degradation and causes accumulation of ubiquitinated proteins on enlarged endosomes 11, 12.

Once internalized cargo has been committed for degradation, conjugated ubiquitin must be recycled and removed by endosomal DUBs such as USP8, which also associate with the ESCRT‐III complex on late endosomes 8, 18. A model was proposed in which USP8 acts further downstream of early endosomes to recycle ubiquitin after endosomal sorting and prior to lysosomal sequestration, suggesting a role in facilitating membrane receptor degradation 14. USP8 thus functions at two stages of plasma membrane receptor trafficking: in early endosomes via ESCRT‐0 interaction or in late endosomes via ESCRT‐III interaction. In this study, we show that regulation of VEGFR2 trafficking and de‐ubiquitination by USP8 impacts on downstream signal transduction and proteolysis.

## Results

### USP8 regulates VEGFR2 trafficking

**Figure 1:**
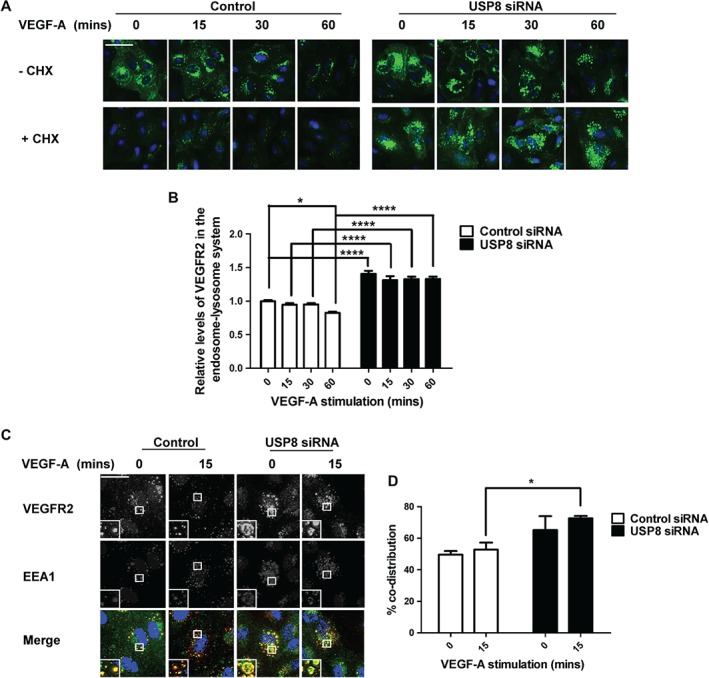
**USP8 is essential for VEGFR2 trafficking.** A) Endothelial cells transfected with non‐targeting or USP8 siRNA, pre‐treated with CHX and stimulated with 25 ng/mL VEGF‐A were fixed and processed for immunofluorescence microscopy using antibodies to VEGFR2 followed by fluorescent species‐specific secondary antibodies (green). Nuclei were stained with DNA‐binding dye, DAPI (blue). Scale bar represents 200 µm. B) Quantification of VEGFR2 levels residing in the endosome‐lysosome system in endothelial cells pre‐treated with CHX and stimulated with VEGF‐A prior to immunofluorescence analysis. C) Endothelial cells transfected with USP8 siRNA and stimulated with 25 ng/mL VEGF‐A for 15 min were fixed and processed for immunofluorescence microscopy using antibodies to VEGFR2 (green) and EEA1 (red) followed by fluorescent species‐specific secondary antibodies. Nuclei were stained with DNA‐binding dye, DAPI (blue). Scale bar represents 70 µm. D) Quantification of co‐distribution between VEGFR2 and EEA1 in endothelial cells treated with non‐targeting or USP8 siRNA and stimulated with VEGF‐A prior to immunofluorescence analysis. Errors bars indicated ±SEM (n ≥ 3); p < 0.05 (*), p < 0.0001 (****).

Previous studies have shown that USP8 depletion causes EGFR accumulation in early endosomes and inhibits downstream degradation due to general defects in endosomal sorting 11. USP8 thus seemed a likely candidate for regulating VEGFR2 trafficking. To test this, we used siRNA duplexes to deplete USP8 in primary human endothelial cells prior to VEGF‐A stimulation and immunofluorescence analysis (Figure 1A). In control cells treated with non‐targeting siRNA, internalized VEGFR2 was detected in punctate structures at early (0–15 min) stages of VEGF‐A stimulation (Figure 1A). After VEGF‐A stimulation for 60 min, VEGFR2 staining was substantially reduced consistent with ligand‐induced degradation (Figure 1A).

However, in USP8‐depleted endothelial cells resting VEGFR2 was already accumulated in enlarged punctate structures (Figure 1A). This pattern of VEGFR2 distribution continued after VEGF‐A stimulation for 60 min suggesting accumulation of VEGFR2 within the endosome‐lysosome system (Figure 1A). Persistence of these enlarged, VEGFR2‐enriched punctate structures following VEGF‐A stimulation indicated perturbed VEGFR2 trafficking and degradation. VEGFR2 accumulation also occurred when cells were treated with individual USP8 siRNAs to limit off‐target effects (Figure S1, Supporting Information). To quantify distribution of mature VEGFR2 in the endosomal pathway, USP8‐depleted endothelial cells were pre‐treated with cycloheximide (CHX) to block protein synthesis followed by VEGF‐A stimulation (Figure 1A). Although the biosynthetic pool of Golgi‐localized VEGFR2 was absent after CHX treatment, mature VEGFR2 still accumulated in USP8‐depleted cells (Figure 1A). Quantification of VEGFR2 residing in the endosome–lysosome system upon CHX treatment revealed VEGFR2 levels were 30% higher in non‐stimulated, USP8‐depleted cells (Figure 1B). In addition, VEGFR2 underwent 20% VEGF‐A‐stimulated degradation in control cells. Contrastingly, high levels of accumulated VEGFR2 persisted in USP8‐depleted cells, undergoing only 5% VEGF‐A‐stimulated degradation (Figure 1B).

To ascertain the nature of these VEGFR2‐enriched structures, USP8‐depleted endothelial cells were stained for VEGFR2 and an early endosome marker, early endosome antigen‐1 (EEA‐1) (Figure 1C). Quantification revealed increased co‐distribution between VEGFR2 and EEA1 before and after VEGF‐A stimulation in USP8‐depleted cells in comparison to control cells (Figure 1D). In addition, USP8‐depleted cells were co‐stained with markers for the plasma membrane (PECAM1), Golgi (TGN46), early endosomes (EEA1), late endosomes (CD63) and lysosomes (LAMP2) (Figure S2A). Quantification revealed that VEGFR2 distribution across these other organelles was minimally affected by USP8 depletion whilst accumulation took place in EEA1‐positive early endosomes (Figure S2B).

VEGFR2 also accumulated in EEA1‐positive early endosomes when cells were treated with individual USP8 siRNAs to limit off‐target effects (Figure S3). In addition, the enlarged VEGFR2‐positive endosomes did not co‐distribute with late endosome marker, CD63, in cells treated with individual USP8 siRNAs (Figure S4). These findings confirm that VEGFR2 accumulates in early endosomes of USP8‐depleted endothelial cells. Thus, USP8 is essential for VEGFR2 trafficking through the endosome–lysosome system.

### USP8 regulates VEGFR2 proteolysis

**Figure 2:**
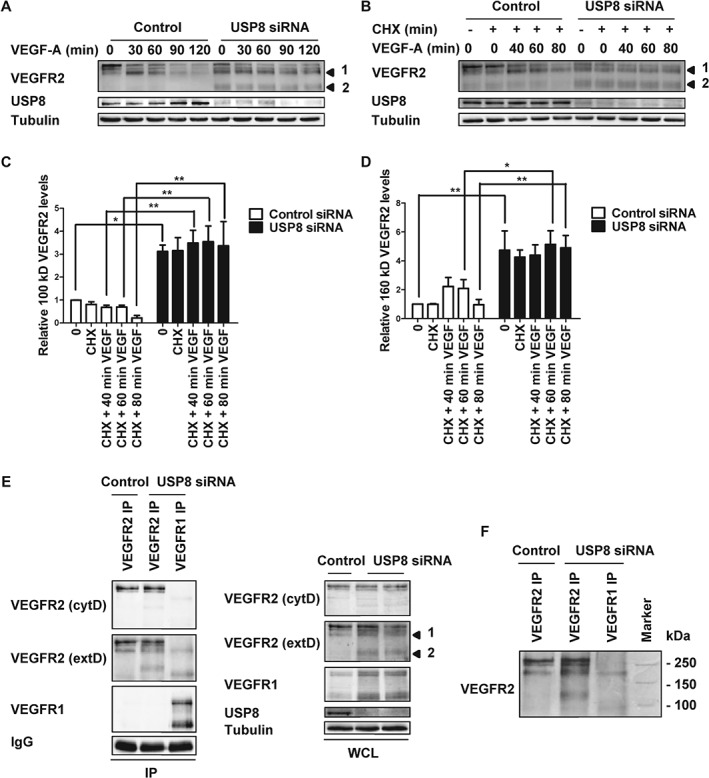
**USP8 depletion promotes generation of a novel 120 kDa VEGFR2 proteolytic cleavage fragment.** Endothelial cells transfected with non‐targeting or USP8 siRNA were treated with 25 ng/mL VEGF‐A (A) or 20 µg/mL CHX and 25 ng/mL VEGF‐A (B), lysed and immunoblotted with antibodies against VEGFR2. Quantification of 120 kDa (C) or 160 kDa (D) VEGFR2 fragment levels in endothelial cells transfected with non‐targeting or USP8 siRNA and treated with 20 µg/mL CHX and 25 ng/mL VEGF‐A. E) VEGFR1 or VEGFR2 were immunoprecipitated from endothelial cells transfected with non‐targeting or USP8 siRNA and immunoblotted with antibodies to the extracellular and cytoplasmic domains of VEGFR2 (F) or run alongside marker ladders to confirm band size. Numbered arrowheads denote the 160 kDa VEGFR2 fragment 1 and the novel 120 kDa VEGFR2 fragment 2. Error bars denote ±SEM (n ≥ 3); p < 0.05 (*), p < 0.01 (**).

**Figure 3:**
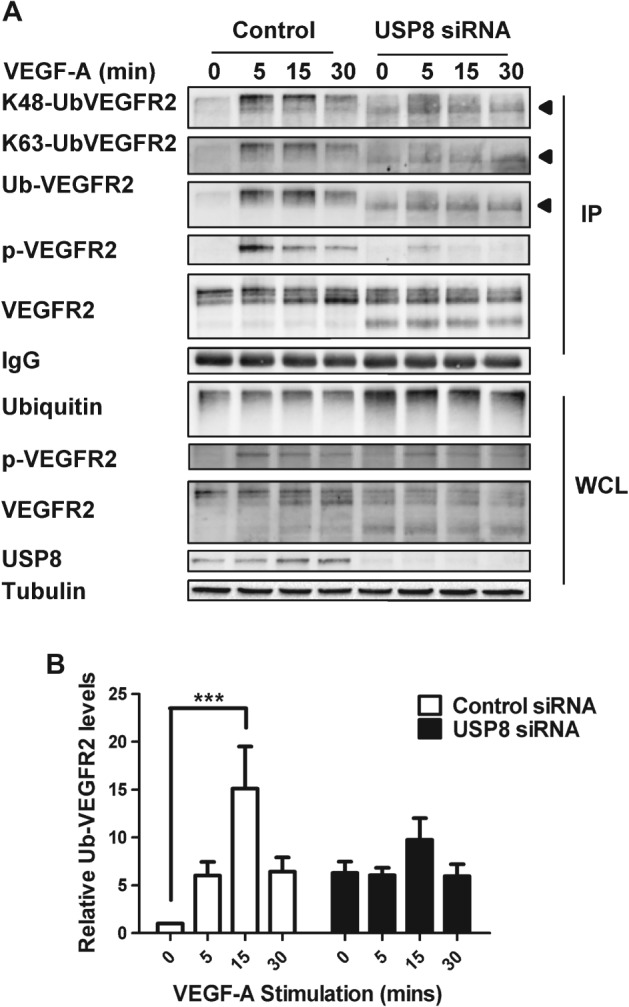
**USP8 regulates VEGFR2 de‐ubiquitination.** A) Endothelial cells transfected with non‐targeting or USP8 siRNA were treated with 25 ng/mL VEGF‐A and lysed. VEGFR2 was immunoprecipitated and immunoblotted for its ubiquitination status using antibodies against K48‐linked polyubiquitin, K63‐linked polyubiquitin and pan‐ubiquitin. Arrowheads denote the species of ubiquitinated VEGFR2 present at higher levels in USP8‐depleted cells. B) Quantification of ubiquitinated VEGFR2 levels in endothelial cells transfected with non‐targeting or USP8 siRNA and treated with 25 ng/mL VEGF‐A. Error bars denote ±SEM (n ≥ 3); p < 0.001 (***).

VEGFR2 proteolysis is regulated by ubiquitination 6, 19. A characteristic feature of VEGFR2 activation is trafficking through the endosome–lysosome system and generation of a transient ∼160 kDa N‐terminal lumenal fragment in endosomes 6. Immunoblotting revealed that the 160 kDa VEGFR2 proteolytic fragment was generated in both control and USP8‐depleted endothelial cells (Figure 2A). However, a novel ∼120 kDa VEGFR2‐derived proteolytic fragment was also produced in USP8‐depleted cells (Figure 2A). Notably, this VEGFR2‐related fragment was also immunoprecipitated from USP8‐depleted cells (Figures 2E, F and 3A).

The novel 120 kDa VEGFR2‐derived proteolytic fragment was present at very low levels in control cells (Figure 2A, B). However, levels increased more than threefold upon USP8 depletion (Figure 2C). Treatment with CHX to block new protein synthesis did not prevent appearance of the 120 kDa fragment, confirming that it is derived from proteolytic cleavage of mature VEGFR2 (Figure 2B, C). USP8‐depleted cells also displayed increased production of the 160 kDa proteolytic fragment (Figure 2D). Thus, the 160 kDa VEGFR2 fragment could be a precursor of the smaller 120 kDa fragment. Alternatively, the 120 kDa fragment may be a unique proteolytic cleavage product that is not cleared efficiently due to a block in forward transport toward the lysosome in USP8‐depleted cells. To resolve the identity of the 120 kDa VEGFR2 fragment VEGFR1 and VEGFR2 were immunoprecipitated from USP8‐depleted cells followed by immunoblot analysis with antibodies to the extracellular and cytoplasmic domains of VEGFR2 (Figure 2E). A VEGFR2 extracellular domain antibody detected all species of VEGFR2 in USP8‐depleted cells. In contrast, the cytoplasmic domain antibody only detected full length immature and mature VEGFR2 (Figure 2E). Thus, similar to the 160 kDa fragment, the 120 kDa VEGFR2 cleavage product is N‐terminal. These findings suggest that USP8 is vital for efficient clearance of VEGFR2 proteolytic cleavage products.

### USP8 regulates VEGFR2 de‐ubiquitination

VEGF‐A binding programs VEGFR2 ubiquitination, endocytosis and proteolysis 4, 6, 19. De‐ubiquitination is required to divert internalized VEGFR2 away from lysosomal degradation and towards recycling 8. Based on the above findings, one possibility is that perturbed VEGFR2 endosome–lysosome trafficking is linked to altered VEGFR2 ubiquitination status in USP8‐depleted cells. To assess VEGFR2 ubiquitination in control and USP8‐depleted cells, VEGFR2 immunoprecipitates were probed by immunoblotting using K48‐ and K63‐linked polyubiquitin‐specific antibodies or a pan‐ubiquitin antibody (Figure 3A). In control cells, VEGFR2 ubiquitination peaked 15 min after VEGF‐A stimulation (Figure 3A). Accumulation of proteolytic cleavage products in USP8‐depleted cells reduced levels of mature VEGFR2 and resulted in lower levels of ubiquitinated full‐length receptor (Figure 3A). However, a lower molecular weight species of ubiquitinated VEGFR2 that corresponds to the 160 kDa proteolytic fragment and represents increased and persistent ubiquitination of this cleavage product was evident in USP8‐depleted cells (Figure 3A, arrowhead). USP8 depletion increased levels of this K48‐ and K63‐linked polyubiquitinated species of VEGFR2. Unlike in control cells, this species of ubiquitinated VEGFR2 was present in non‐stimulated cells and persisted over a time course of VEGF‐A stimulation (Figure 3A). Quantification of immunoblot data showed that whereas ligand‐stimulated VEGFR2 ubiquitination displayed a characteristic peak and decline, under conditions of USP8 depletion VEGFR2 ubiquitination persisted (Figure 3B). One possibility is that reduced de‐ubiquitination of accumulated VEGFR2 in USP8‐depleted cells increased susceptibility to proteolysis. Thus, proteolytic cleavage products remain ubiquitinated and accumulate in early endosomes. These data suggest that USP8 is a key regulator of VEGFR2 de‐ubiquitination.

### VEGF‐A‐stimulated VEGFR2 signal transduction is perturbed by USP8 depletion

**Figure 4:**
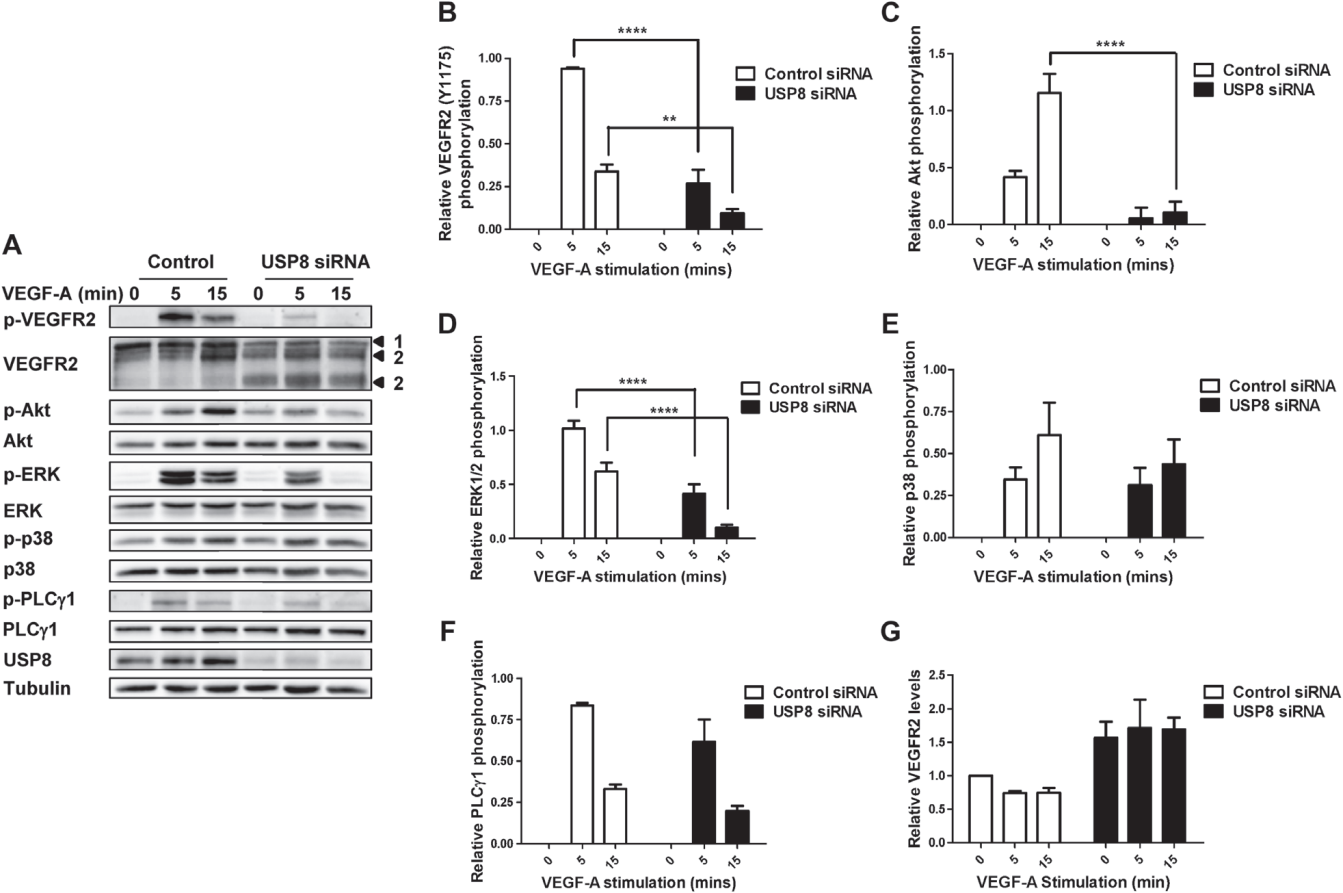
**USP8 depletion inhibits VEGF‐A‐stimulated VEGFR2 signal transduction.** A) Endothelial cells transfected with non‐targeting or USP8 siRNA were treated with 25 ng/mL VEGF‐A, lysed and immunoblotted for phospho‐VEGFR2 (Y1175), phospho‐Akt (S473), phospho‐ERK1/2 (T202/Y204) phospho‐p38 MAPK (T180/Y182) and phospho‐PLCγ1 (Y783). Quantification of phospho‐VEGFR2 (B), phospho‐Akt (C), phospho‐ERK1/2 (D), phospho‐p38 MAPK (E), phopho‐PLCγ1 (F) and VEGFR2 (G) levels in endothelial cells transfected with non‐targeting or USP8 siRNA. Numbered arrowheads denote mature VEGFR2 (1) and proteolytic VEGFR2 fragments (2). Errors bars indicated ±SEM (n ≥ 3); p < 0.01 (**), p < 0.0001 (****).

VEGF‐A stimulates multiple signal transduction pathways in endothelial cells 20 that regulate many cellular responses 21. Furthermore, VEGF‐A‐stimulated signal transduction is dependent on positional location of VEGFR2 at the plasma membrane or within endosome‐related compartments 6, 20, 22. However, the role of the ubiquitination/de‐ubiquitination cycle in RTK signal transduction is unclear. Perturbed VEGFR2 endosomal trafficking caused by USP8 depletion could modulate endosome‐linked signal transduction. Control or USP8‐depleted endothelial cells were subjected to a time course of VEGF‐A stimulation followed by immunoblot analysis to assess VEGFR2 activation and downstream signal transduction (Figure 4A). VEGF‐A binding causes autophosphorylation of VEGFR2 cytoplasmic residue Y1175, creating a key‐binding site for downstream effectors 20. Quantification of immunoblot analysis revealed VEGFR2‐pY1175 levels were ∼60% reduced in USP8‐depleted endothelial cells (Figure 4B). VEGF‐A‐stimulated activation of the master regulator and serine/threonine protein kinase, Akt, was ∼90% reduced in USP8‐depleted cells (Figure 4C). In addition, VEGF‐A‐stimulated activation of the mitogen activated protein kinase (MAPK) pathway involving ERK1/2 was ∼60% reduced in USP8‐depleted cells (Figure 4D). Interestingly, USP8 depletion did not significantly affect plasma membrane‐associated phosphorylation and activation of p38 MAPK or PLCγ1 (Figure 4E, F). USP8 activity thus modulates VEGF‐A‐stimulated Akt and ERK1/2 activation but does not affect other VEGFR2‐associated signal transduction pathways.

Levels of mature, full‐length VEGFR2 appear reduced in USP8‐depleted cells (Figure 4A) yet VEGFR2 is accumulated in early endosomes (Figure 1A). VEGFR2 proteolysis in early endosomes generates 120 and 160 kDa N‐terminal fragments. Proteolytic cleavage products of mature VEGFR2 accumulate in early endosomes of USP8‐depleted cells after failure to reach a degradative compartment thus causing an overall increase in total VEGFR2 levels (Figure 4G).

### VEGFR2 plasma membrane dynamics

**Figure 5:**
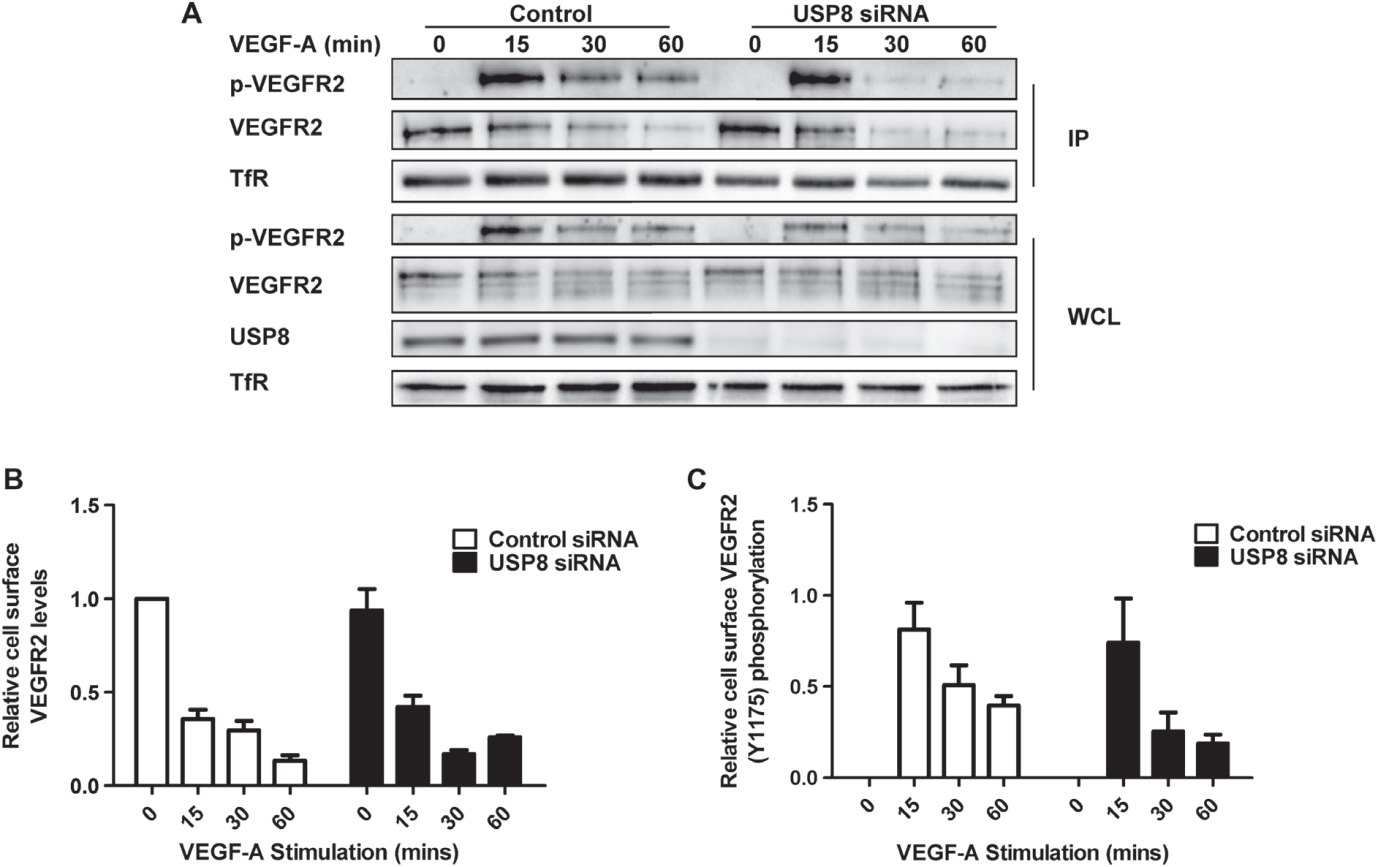
**USP8 depletion does not affect VEGFR2 levels or activation at the plasma membrane.** A) Endothelial cells transfected with non‐targeting or USP8 siRNA were treated with 25 ng/mL VEGF‐A, cell surface biotinylated and lysed. Biotinylated proteins were isolated and immunoblotted using antibodies against phospho‐VEGFR2 (Y1175) and VEGFR2. Quantification of cell surface VEGFR2 (B) or phospho‐VEGFR2 (C) levels over a time course of VEGF‐A stimulation in endothelial cells transfected with non‐targeting or USP8 siRNA. Error bars denote ±SEM (n ≥ 3).

In USP8‐depleted endothelial cells non‐stimulated VEGFR2 displayed accumulation in early endosomes (Figure 1A). Reduced VEGFR2 availability at the plasma membrane could thus diminish response to exogenously added VEGF‐A. To investigate this possibility, we used cell surface biotinylation to compare plasma membrane VEGFR2 pools in control and USP8‐depleted cells (Figure 5A). Quantification revealed little difference in non‐stimulated, plasma membrane VEGFR2 levels between control and USP8‐depleted cells (Figure 5B). Furthermore, USP8 depletion had little effect on relative VEGFR2 levels at the plasma membrane over a 60 min time course of VEGF‐A stimulation. Thus, reduced plasma membrane VEGFR2 availability did not account for diminished VEGF‐A‐dependent Akt and ERK1/2 activation (Figures 4C, D and 5B). Monitoring peak VEGFR2‐Y1175 phosphorylation at the plasma membrane also revealed little difference between control and USP8‐depleted cells (Figure 5C). Thus, inhibition of VEGFR2 phosphorylation (Figure 4B) in USP8‐depleted cells likely represents an effect on the total VEGFR2 pool which continues to be phosphorylated after internalization. These findings suggest that USP8 does not regulate plasma membrane VEGFR2 levels and subsequent activation at the cell surface.

**Figure 6:**
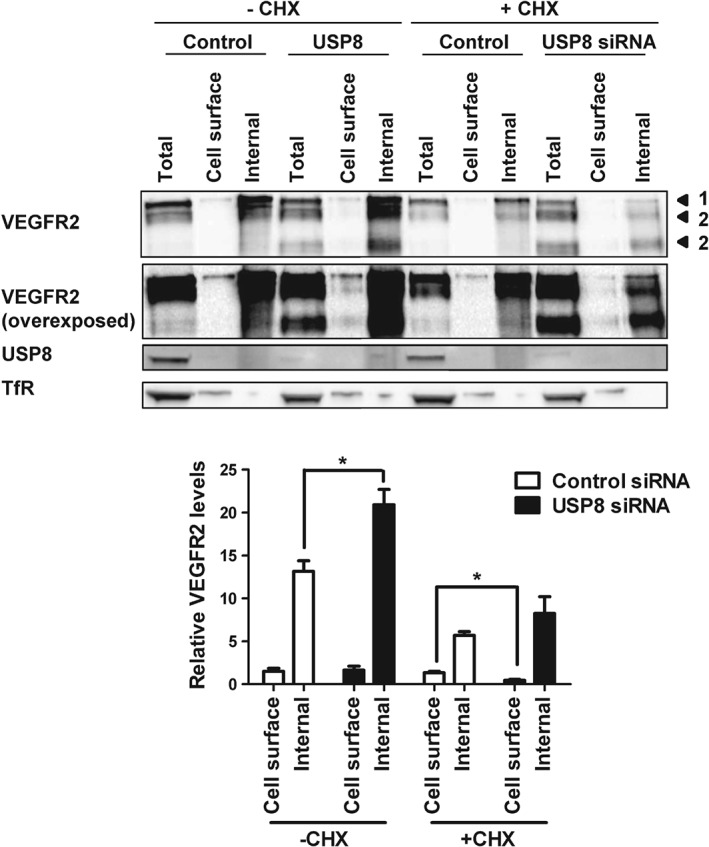
**Plasma membrane VEGFR2 is replenished by newly synthesized receptor in USP8‐depleted cells.** A) Endothelial cells transfected with non‐targeting or USP8 siRNA were treated with 10 µg/mL CHX, cell surface biotinylated and lysed. Biotinylated proteins were isolated and the internal VEGFR2 pool immunoprecipitated from the supernatant followed by immunoblot analysis using antibodies against VEGFR2. B) Quantification of VEGFR2 levels in cells transfected with non‐targeting or USP8 siRNA and treated with 10 µg/mL CHX. Numbered arrowheads denote mature VEGFR2 (1) and proteolytic VEGFR2 fragments (2). Error bars denote ±SEM (n ≥ 3); p < 0.05 (*).

VEGFR2 accumulates in early endosomes in USP8‐depleted cells, yet, plasma membrane levels are unaffected (Figures 1B, 5B). One possibility is that plasma membrane VEGFR2 is replenished by newly synthesized receptor. To test this, we used biotinylation to compare plasma membrane and internal VEGFR2 pools in control and USP8‐depleted cells treated with CHX. Biotinylated plasma membrane proteins were isolated and internal VEGFR2 was immunoprecipitated followed by immunoblot analysis (Figure 6A). Quantification revealed that levels of internal VEGFR2 were significantly higher in USP8‐depleted cells after accumulation in early endosomes, while plasma membrane VEGFR2 was unaffected. Upon CHX treatment, cell surface VEGFR2 levels were significantly reduced in USP8‐depleted cells (Figure 6B). Thus, VEGFR2 that accumulates in early endosomes following cleavage into proteolytic fragments is replenished at the cell surface by the biosynthetic pool of receptor.

## Discussion

**Figure 7:**
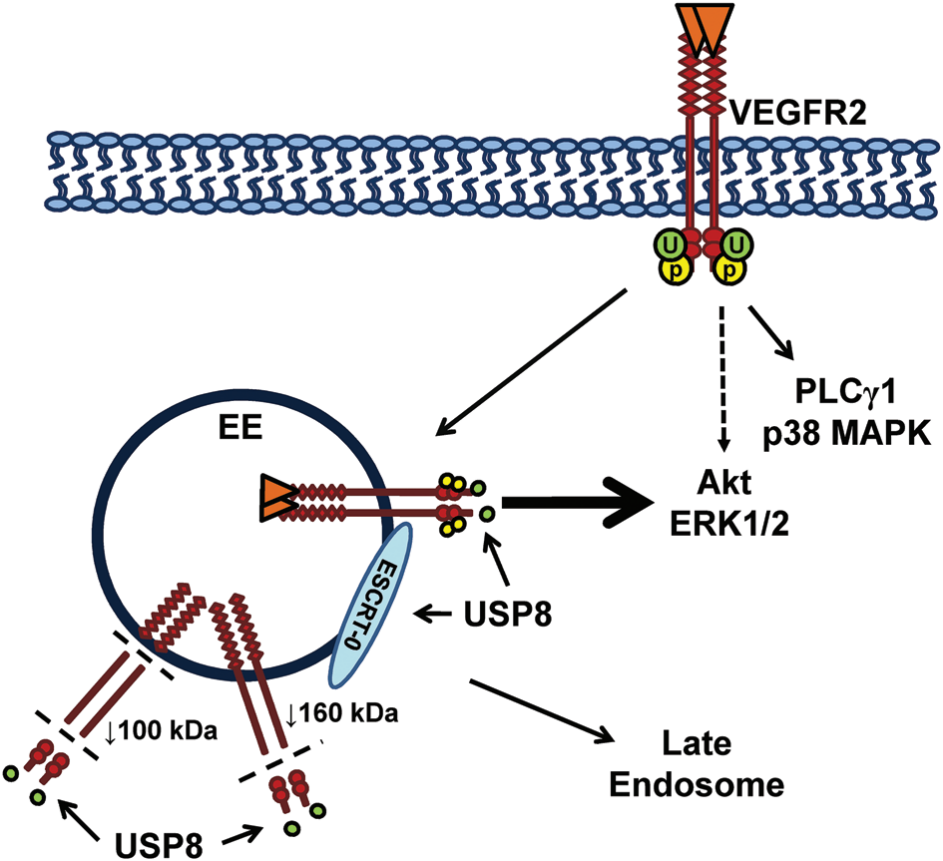
**Regulation of VEGFR2 trafficking, signaling and proteolysis by USP8.** Sustained VEGFR2 residence and ubiquitination in early endosomes leads to proteolysis that generates 160 and 120 kDa VEGFR2 fragments in USP8‐depleted cells. The 160 kDa fragment remains ubiquitinated and both VEGFR2 cleavage products accumulate in early endosomes due to an indirect effect of USP8 depletion on the ESCRT‐0 trafficking machinery. Both reduced de‐ubiquitination and enhanced proteolysis of accumulated VEGFR2 down‐regulate endosome‐linked signal transduction.

In this study, we show that USP8 plays a key role in VEGFR2 trafficking and proteolysis in the endosome–lysosome system. In this model, sustained VEGFR2 ubiquitination in the absence of USP8 promotes generation of a novel 120 kDa VEGFR2 proteolytic fragment (Figure 7). In addition, USP8‐mediated de‐ubiquitination and trafficking of VEGFR2 is linked to spatio‐temporal control of VEGF‐A‐stimulated signal transduction (Figure 7).

Our conclusions are based on four lines of evidence. First, USP8 depletion leads to VEGFR2 accumulation within an endosome‐like compartment that also contains the early endosome marker, EEA‐1. Second, the appearance of a novel ∼120 kDa VEGFR2‐derived proteolytic fragment indicates the existence of a sequence of compartmental‐specific machinery that regulates VEGFR2 proteolysis. Depletion of USP8 is marked by aberrant VEGFR2 ubiquitination and turnover, hallmarks of perturbed ubiquitination and sorting in the endosome–lysosome system. Whereas ERK1/2 and Akt signaling is inhibited by USP8 depletion, other signal transduction pathways are unaffected. The temporal sequence of events in this pathway suggests that ubiquitination and de‐ubiquitination are closely associated with endosomes and have major implications for cellular decision making processes.

Endosomal DUBs are postulated to remove ubiquitin chains on membrane ‘cargo’ and thus prevent terminal degradation in lysosomes. Previous work reported that USP8 depletion severely inhibits EGFR degradation 11. Ubiquitinated EGFR accumulated in EEA1‐positive endosomes caused by failure to reach a terminal degradative compartment 11. Other work also supported the view that EGFR down‐regulation is USP8‐dependent 12. Our study now shows that a different RTK, VEGFR2, accumulates in early endosomes of USP8‐depleted cells. It is likely that both full‐length and partially proteolysed VEGFR2 accumulates in these endosomes upon USP8 depletion.

Ubiquitin‐linked enzyme activity of DUBs plays vital roles in RTK trafficking 23. USP8 modulates EGFR trafficking by regulating STAM de‐ubiquitination on early endosomes 11. STAM protection from proteasomal degradation facilitates forward movement of ubiquitinated receptors through the endosome–lysosome system. USP8 depletion causes almost complete STAM degradation and hinders further trafficking of cargo proteins 14. Our studies also lead to the conclusion that USP8 regulates endosomal machinery (e.g. ESCRT‐0) that is essential for onward trafficking of activated VEGFR2 through the endosome–lysosome system.

VEGFR2 localization in early endosomes is linked to subsequent 26S proteasome‐dependent proteolysis and production of a 160 kDa proteolytic fragment 6. Furthermore, production of the 160 kDa fragment has been linked to down‐regulation of VEGFR2 endosomal signaling 6. This study shows that USP8 depletion is linked to generation of a novel 120 kDa proteolytic fragment derived from mature plasma membrane VEGFR2. We postulate that the 230 kDa mature VEGFR2 protein undergoes sequential proteolysis in the endosomal network into 160 and 120 kDa extracellular domain fragments. These VEGFR2 fragments could also be individual cleavage products that remain trapped in early endosomes and inaccessible to lysosomal degradation due to disruption of ESCRT‐0 components and endosome–lysosome delivery upon USP8 depletion. Another possibility is that an imbalance in the levels of hydrolases as a result of USP8 depletion promotes increased VEGFR2 processing and release of the extracellular domain within early endosomes.

USP8 regulates de‐ubiquitination of EGFR/ErbB1 and ErbB2 11, 15, 24. We now provide evidence that USP8 de‐ubiquitinates VEGFR2. Whereas VEGFR2 ubiquitination showed a ligand‐dependent peak and decline in control cells, ubiquitination of the 160 kDa VEGFR2 proteolytic fragment persisted over a time course of VEGF‐A stimulation in USP8‐depleted cells.

Reduced VEGFR2 de‐ubiquitination upon USP8 depletion did not promote increased lysosomal degradation due to the indirect effect of USP8 depletion on the trafficking machinery causing accumulation of ubiquitinated VEGFR2 in early endosomes. This ubiquitinated VEGFR2 was susceptible to proteolysis which reduced levels of full‐length receptor but caused accumulation of cleavage products in the endosome–lysosome system. VEGFR2 proteolytic fragments of 160 and 120 kDa thus accumulated in early endosomes causing an overall increase in total VEGFR2 levels.

Lys48 (K48)‐linked polyubiquitin chains are associated with proteasomal degradation while Lys63 (K63)‐linked chains are associated with membrane receptor trafficking and targeting in the endosome–lysosome system 25, 26. Our finding that increased levels of both K48‐ and K63‐linked polyubiquitin are present on VEGFR2 upon USP8 depletion agrees with other studies that USP8 removes both such polyubiquitin chains from protein susbtrates 14.

VEGFR2 signal transduction is postulated to be location dependent with different outcomes at the plasma membrane versus endosomes 22. A requirement for USP8 was evident in VEGF‐A‐stimulated ERK1/2 and Akt activation but not in p38 MAPK and PLCγ1 activation. This outcome could be linked to perturbed VEGFR2 localization in endosomes while plasma membrane dynamics are unaffected due to replenishment from the biosynthetic pool in USP8‐depleted cells. Other studies have also linked activated VEGFR2 residence in endosomes to ERK1/2 6, 20, 22, 27, 28 and Akt 20, 22, 27, 29 activation. One possible explanation is that activated VEGFR2 must undergo USP8‐mediated de‐ubiquitination on early endosomes as a pre‐requisite for engagement with ERK1/2 and Akt‐linked signal transduction machinery

Our model proposes that sustained VEGFR2 residence and ubiquitination in early endosomes leads to proteolysis that generates 160 and 120 kDa VEGFR2 fragments in USP8‐depleted cells (Figure 7). Although the 160 kDa fragment remains ubiquitinated, both VEGFR2 cleavage products accumulate in early endosomes because of the indirect effect of USP8 depletion on trafficking machinery. Both reduced de‐ubiquitination and enhanced proteolysis of accumulated VEGFR2 down‐regulate endosome‐linked signal transduction (Figure 7).

USP8 is required for efficient VEGFR2 trafficking, de‐ubiquitination, signal transduction and proteolysis. This study emphasizes plasma membrane receptor ubiquitination and de‐ubiquitination is more complex than a simple system for mediating degradation in lysosomes. Modulation of VEGFR2 ubiquitination status could provide a new and alternative therapeutic strategy to target disease states that display aberrant angiogenesis.

## Materials and methods

### Cell culture and materials

Human umbilical vein endothelial cells (HUVECs) were isolated and cultured as previously described 30, 31. The following reagents were purchased: goat anti‐VEGFR2 (R&D Systems), rabbit anti‐phospho‐VEGFR2 (Y1175), rabbit antibodies to native and phosphorylated c‐Akt (S473), PLCγ1 (Y783) and p38 (T180/Y182), rabbit anti‐ERK1/2, mouse anti‐phospho‐ERK1/2 (T202, Y204), rabbit anti‐USP8 (Cell Signaling Technologies), rabbit anti‐TGN46, mouse anti‐EEA1 (BD Biosciences), mouse anti‐CD63 (AbCam), mouse anti‐α‐tubulin, mouse anti‐PECAM1, mouse anti‐transferrin receptor (TfR) (Santa Cruz Biotechnology), mouse FK2 anti‐ubiquitin (Affiniti Research Products), rabbit Apu2 anti‐K48‐linked ubiquitin (Millipore), rabbit Apu3 anti‐K63‐linked ubiquitin (Millipore), HRP‐conjugated secondary antibodies (PerBio Sciences), AlexaFluor‐conjugated secondary antibodies (Invitrogen), endothelial cell growth medium (ECGM) (PromoCell), non‐targeting and USP8 siGENOME SMARTpool siRNA duplexes (Dharmacon, GE Healthcare) and recombinant human VEGF‐A_165a_ (Genetech Inc.).

### Immunoblotting and immunofluorescence

HUVECs were serum starved in MCDB131 (Life Technologies) for 2 h prior to stimulation with 25 ng/mL VEGF‐A_165a_ for various times and lysed for immunoblotting or immunoprecipitation analyses. In some experiments, cells were pre‐treated with 10 µg/mL CHX (2 h) prior to VEGF‐A stimulation or with 20 µg/mL CHX at the same time as VEGF‐A stimulation. Lysate preparation and immunoblot analysis were performed as previously described 30, 31. For immunofluorescence analysis, cells were serum starved and pre‐treated with 10 µg/mL CHX for 2 h prior to stimulation with VEGF‐A before being fixed and processed as previously described 30, 31. Images were acquired using an Evos‐fI inverted digital microscope (Life Technologies) or a wide‐field deconvolution microscope DeltaVision (Applied Precision Inc.). Relative co‐distribution was quantified using NIH Image J (http://rsb.info.nih.gov/ij/).

### Immunoprecipitation

HUVECs were serum starved for 2 h prior to VEGF‐A stimulation, lysed in buffer (150 mm NaCl, 50 mm Tris–HCl, pH 7.4, 0.1% SDS, 0.5% sodium deoxycholate, 2 mm EDTA, 1% NP‐40, 50 mm NaF, 1 mm PMSF and 10 mm iodoacetamide) and incubated with 1 µg/mL goat anti‐VEGFR2 for 2 h. Immune complexes were captured using Protein G‐agarose beads before SDS‐PAGE and immunoblot analyses.

### Cell surface biotinylation

HUVECs were serum starved for 2 h prior to VEGF‐A stimulation, washed in ice‐cold PBS and cell surface biotinylation carried out by incubation with 0.25 mg/mL biotin (in 2 mm CaCl_2_, 2 mm MgCl_2_, PBS) for 45 min. Cells were then washed in TBS to quench biotinylation and lysed in buffer (1% NP‐40, 50 mm Tris–HCl, pH 7.5, 150 mm NaCl, 1 mm PMSF). Biotinylated cell surface proteins were isolated using NeutraAvidin‐agarose beads (ThermoFisher) before SDS‐PAGE and immunoblotting.

### siRNA reverse transfection

HUVECs were reverse transfected in 6‐ or 96‐well plates with siRNA duplexes as follows.

20 nm non‐targeting control siRNA:

5′‐UAAGGCUAUGAAGAGAUAC‐3′

5′‐AUGUAUUGGCCUGUAUUAG‐3′

5′‐AUGAACGUGAAUUGCUCAA‐3′

5′‐UGGUUUACAUGUCGACUAA‐3′

20 nm USP8 siRNA:

5′‐UGAAAUACGYGACUGUUUA‐3′

5′‐GGACAGGACAGUAUAGAUA‐3′

5′‐AAAUAAAGCUCAACGAGAA‐3′

5′‐GGCAAGCCAUUUAAGAUUA‐3′

All siRNA duplexes were from ThermoFisher and used according to the manufacturer's instructions. Endothelial cells were incubated for 6 h with siRNA duplexes using a previously described lipid‐based transfection protocol 30. After 72 h, cells were processed for lysis and immunoblotting as previously described.

### Statistical analysis

Statistical analysis was performed using a one‐way analysis of variance (ANOVA) and Tukey's post‐test analysis for multiple comparisons or two‐way ANOVA followed by the Bonferroni multiple comparison test using GraphPad Prism software (La Jolla). Significant differences between control and test groups were evaluated with p‐values less than 0.05 (*), 0.01 (**), 0.001 (***) and 0.0001 (****) indicated on the graphs. Error bars on graphs denote ± standard error of mean (SEM) of results from at least three independent experiments.

## Supporting information


**Figure S1**. **Individual USP8 siRNAs perturb VEGFR2 trafficking**. A) Endothelial cells transfected with non‐targeting or individual USP8 siRNAs and stimulated with 25 ng/mL VEGF‐A were fixed and processed for immunofluorescence microscopy using antibodies to VEGFR2 followed by fluorescent species‐specific secondary antibodies (green). Nuclei were stained with DNA‐binding dye, DAPI (blue). Scale bar represents 200 µm. B) To confirm USP8 depletion, endothelial cells transfected with non‐targeting or individual USP8 siRNAs were lysed and immunoblotted with antibodies against USP8.
**Figure S2**. **Cellular distribution of VEGFR2 in USP8‐depleted cells.** A) Endothelial cells transfected with non‐targeting or USP8 siRNA were fixed and processed for immunofluorescence microscopy using antibodies to VEGFR2 (green) and PECAM1, LAMP2, TGN46, EEA1 or CD63 (red) followed by species‐specific secondary antibodies. Nuclei were stained with DNA‐binding dye, DAPI (blue). Scale bar represents 70 µm. B) Quantification of co‐distribution between VEGFR2 and cellular markers in endothelial cells treated with control or USP8 siRNA. Error bars denote ±SEM (n ≥ 3), p < 0.05 (*).
**Figure S3**. **Individual USP8 siRNAs cause VEGFR2 accumulation in early endosomes.** Endothelial cells transfected with individual USP8 siRNAs and stimulated with 25 ng/mL VEGF‐A for 15 min were fixed and processed for immunofluorescence microscopy using antibodies to VEGFR2 (green) and EEA1 (red) followed by fluorescent species‐specific secondary antibodies. Nuclei were stained with DNA‐binding dye, DAPI (blue). Arrows indicate enlarged VEGFR2‐positive early endosomes. Scale bar represents 70 µm.
**Figure S4**. **Individual USP8 siRNAs do not cause VEGFR2 accumulation in late endosomes.** Endothelial cells transfected with individual USP8 siRNAs and stimulated with 25 ng/mL VEGF‐A for 15 min were fixed and processed for immunofluorescence microscopy using antibodies to VEGFR2 (green) and CD63 (red) followed by fluorescent species‐specific secondary antibodies. Nuclei were stained with DNA‐binding dye, DAPI (blue). Arrows indicate enlarged VEGFR2‐positive early endosomes. Scale bar represents 70 µm.
