# Supplementary material for: VEGFR2 Trafficking, Signaling and Proteolysis is Regulated by the Ubiquitin Isopeptidase USP8
Source: Traffic. 2015 Dec 2;17(1):53–65. doi: 10.1111/tra.12341 (PMC4832373; doi:10.1111/tra.12341)
Supplement: Supplementary file 1 — Figure S1. Individual USP8 siRNAs perturb VEGFR2 trafficking. A) Endothelial cells transfected with non‐targeting or individual USP8 siRNAs and stimulated with 25 ng/mL VEGF‐A were fixed and processed for immunofluorescence microscopy using antibodies to VEGFR2 followed by fluorescent species‐specific secondary antibodies (green). Nuclei were stained with DNA‐binding dye, DAPI (blue). Scale bar represents 200 µm. B) To confirm USP8 depletion, endothelial cells transfected with non‐targeting or individual USP8 siRNAs were lysed and immunoblotted with antibodies against USP8. Figure S2. Cellular distribution of VEGFR2 in USP8‐depleted cells. A) Endothelial cells transfected with non‐targeting or USP8 siRNA were fixed and processed for immunofluorescence microscopy using antibodies to VEGFR2 (green) and PECAM1, LAMP2, TGN46, EEA1 or CD63 (red) followed by species‐specific secondary antibodies. Nuclei were stained with DNA‐binding dye, DAPI (blue). Scale bar represents 70 µm. B) Quantification of co‐distribution between VEGFR2 and cellular markers in endothelial cells treated with control or USP8 siRNA. Error bars denote ±SEM (n ≥ 3), p < 0.05 (*). Figure S3. Individual USP8 siRNAs cause VEGFR2 accumulation in early endosomes. Endothelial cells transfected with individual USP8 siRNAs and stimulated with 25 ng/mL VEGF‐A for 15 min were fixed and processed for immunofluorescence microscopy using antibodies to VEGFR2 (green) and EEA1 (red) followed by fluorescent species‐specific secondary antibodies. Nuclei were stained with DNA‐binding dye, DAPI (blue). Arrows indicate enlarged VEGFR2‐positive early endosomes. Scale bar represents 70 µm. Figure S4. Individual USP8 siRNAs do not cause VEGFR2 accumulation in late endosomes. Endothelial cells transfected with individual USP8 siRNAs and stimulated with 25 ng/mL VEGF‐A for 15 min were fixed and processed for immunofluorescence microscopy using antibodies to VEGFR2 (green) and CD63 (red) followed by fluorescent species‐specifi [file TRA-17-53-s001.docx]

**VEGFR2 trafficking, signaling and proteolysis is regulated by the ubiquitin isopeptidase USP8**

**Gina A. Smith^1^, Gareth W. Fearnley^1^, Izma A. Zani^1^, Stephen B. Wheatcroft^2^, Darren C. Tomlinson^3^,**

**Michael A. Harrison^4^, Sreenivasan Ponnambalam^1*^**

**Supplemental Materials**

**
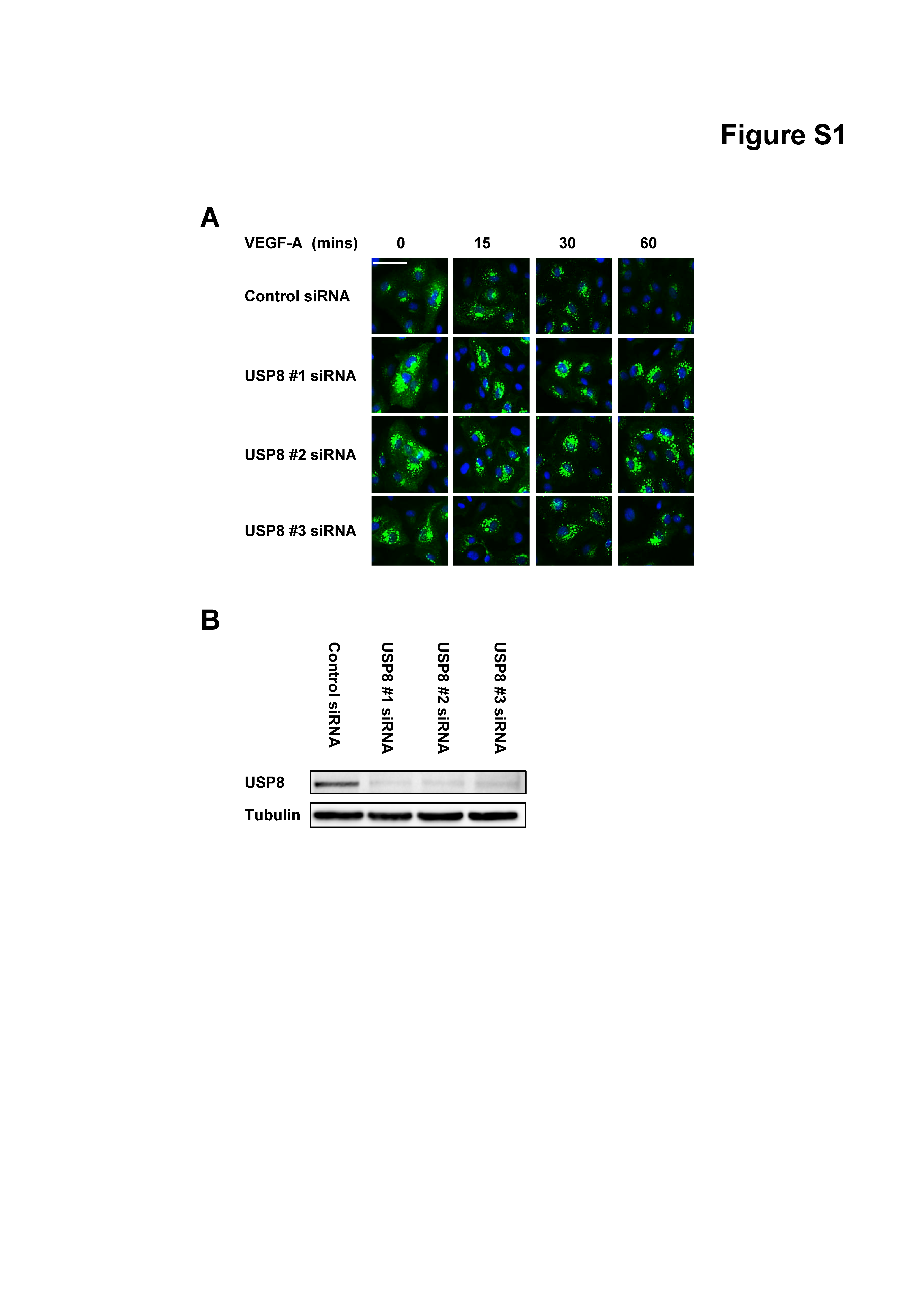
**

**Figure S1. Individual USP8 siRNAs perturb VEGFR2 trafficking.** (A) Endothelial cells transfected with non-targeting or individual USP8 siRNAs and stimulated with 25 ng/ml VEGF-A were fixed and processed for immunofluorescence microscopy using antibodies to VEGFR2 followed by fluorescent species-specific secondary antibodies (green). Nuclei were stained with DNA-binding dye, DAPI (blue). Scale bar represents 200 μm. (B) To confirm USP8 depletion, endothelial cells transfected with non-targeting or individual USP8 siRNAs were lysed and immunoblotted with antibodies against USP8.

**
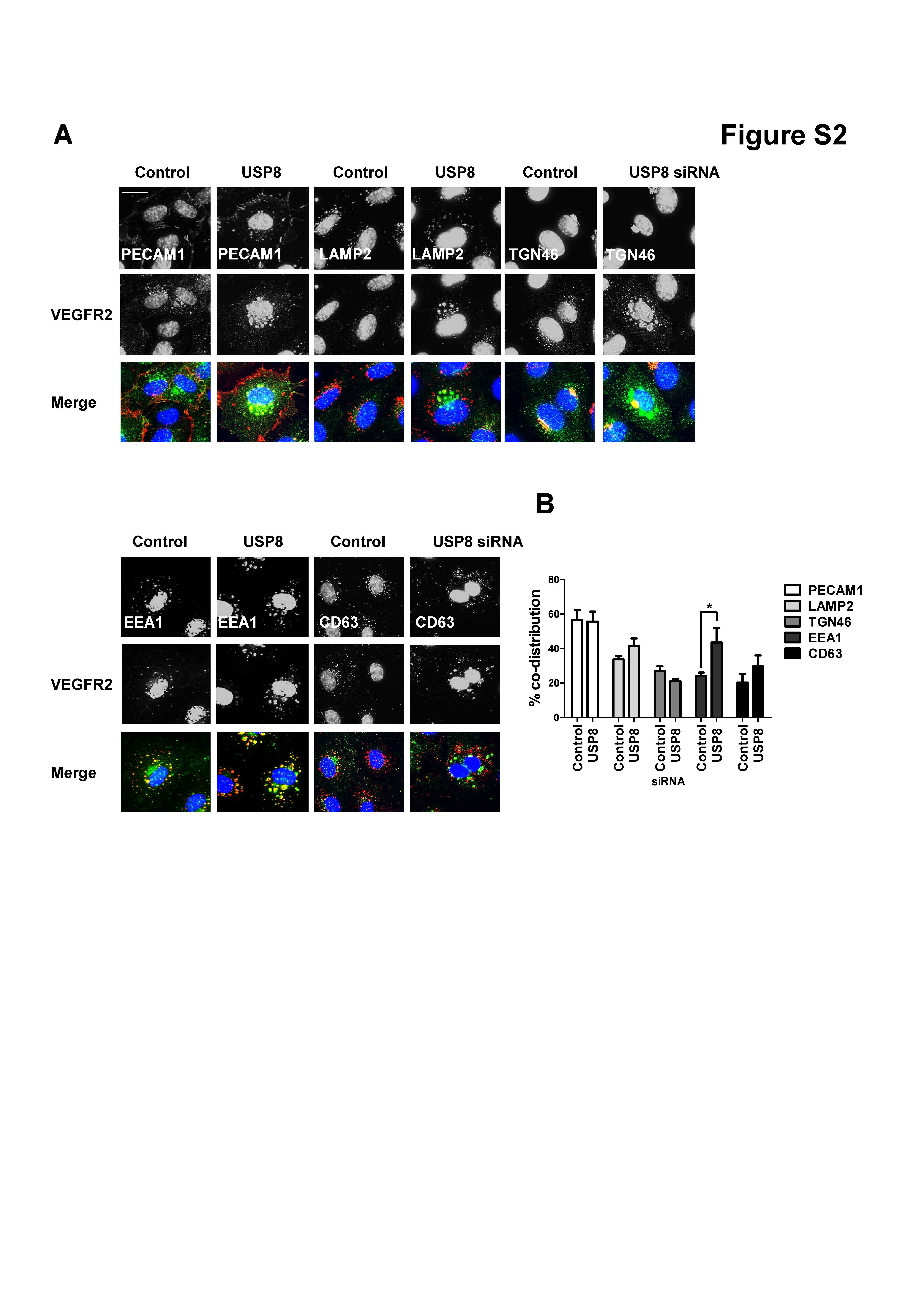
**

**Figure S2. Cellular distribution of VEGFR2 in USP8-depleted cells.** (A) Endothelial cells transfected with non-targeting or USP8 siRNA were fixed and processed for immunofluorescence microscopy using antibodies to VEGFR2 (green) and PECAM1, LAMP2, TGN46, EEA1 or CD63 (red) followed by species-specific secondary antibodies. Nuclei were stained with DNA-binding dye, DAPI (blue). Scale bar represents 70 μm. (B) Quantification of co-distribution between VEGFR2 and cellular markers in endothelial cells treated with control or USP8 siRNA. Error bars denote ±SEM (n≥3). *p*<0.05 (*).

**
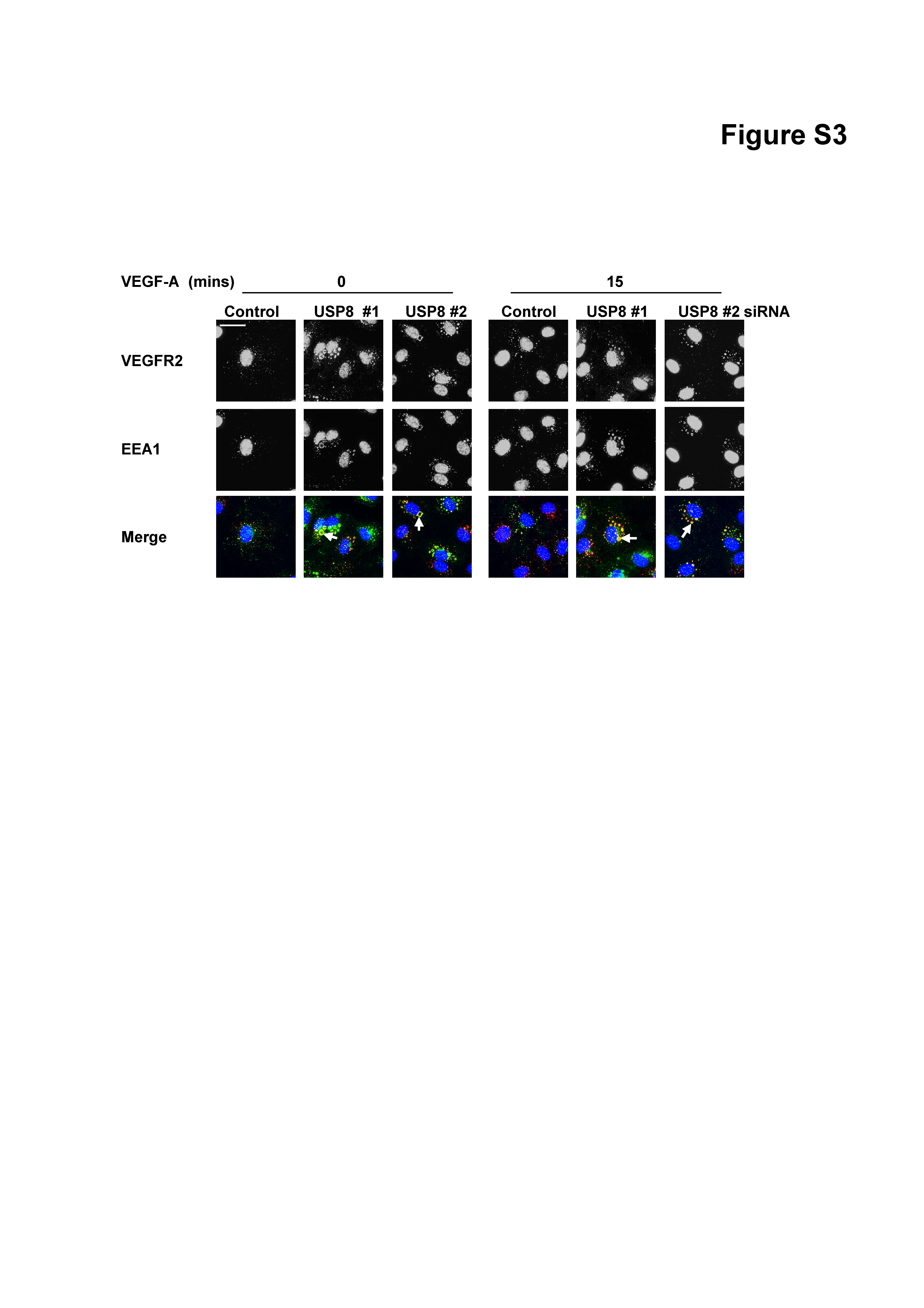
**

**Figure S3. Individual USP8 siRNAs cause VEGFR2 accumulation in early endosomes.** Endothelial cells transfected with individual USP8 siRNAs and stimulated with 25 ng/ml VEGF-A for 15 min were fixed and processed for immunofluorescence microscopy using antibodies to VEGFR2 (green) and EEA1 (red) followed by fluorescent species-specific secondary antibodies. Nuclei were stained with DNA-binding dye, DAPI (blue). Arrows indicate enlarged VEGFR2-positive early endosomes. Scale bar represents 70 μm.

**
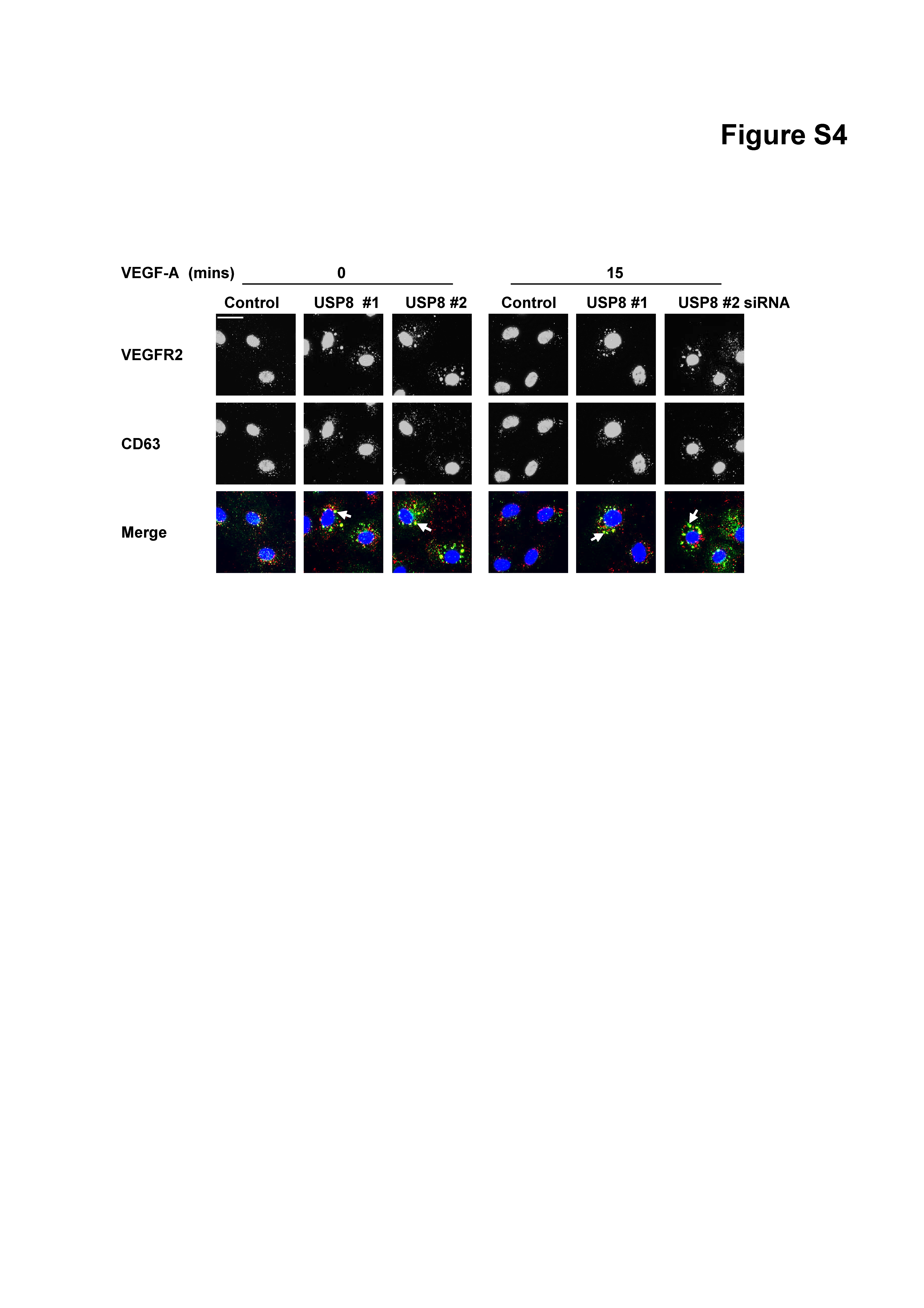
**

**Figure S4. Individual USP8 siRNAs do not cause VEGFR2 accumulation in late endosomes.** Endothelial cells transfected with individual USP8 siRNAs and stimulated with 25 ng/ml VEGF-A for 15 min were fixed and processed for immunofluorescence microscopy using antibodies to VEGFR2 (green) and CD63 (red) followed by fluorescent species-specific secondary antibodies. Nuclei were stained with DNA-binding dye, DAPI (blue). Arrows indicate enlarged VEGFR2-positive early endosomes. Scale bar represents 70 μm.
